# Tetrasubstituted Imidazolium Salts as Potent Antiparasitic Agents against African and American Trypanosomiases

**DOI:** 10.3390/molecules23010177

**Published:** 2018-01-16

**Authors:** Ouldouz Ghashghaei, Nicola Kielland, Marc Revés, Martin C. Taylor, John M. Kelly, Ornella Di Pietro, Diego Muñoz-Torrero, Belén Pérez, Rodolfo Lavilla

**Affiliations:** 1 Laboratory of Organic Chemistry, Faculty of Pharmacy and Food Sciences, University of Barcelona, Barcelona Science Park, Baldiri Reixac 10-12, 08028 Barcelona, Spain; ouldouz.ghashghaei@gmail.com (O.G.); nicola.kielland@gmail.com (N.K.); revesmarc@gmail.com (M.R.); 2 Department of Pathogen Molecular Biology, London School of Hygiene and Tropical Medicine, London WC1E 7HT, UK; Martin.Taylor@lshtm.ac.uk (M.C.T.); john.kelly@lshtm.ac.uk (J.M.K.); 3 Laboratory of Pharmaceutical Chemistry (CSIC Associated Unit), Faculty of Pharmacy and Food Sciences, and Institute of Biomedicine (IBUB), University of Barcelona, 08028 Barcelona, Spain; od265@cam.ac.uk (O.D.P.); dmunoztorrero@ub.edu (D.M.-T.); 4 Department of Pharmacology, Therapeutics and Toxicology, Institute of Neurosciences, Autonomous University of Barcelona, 08193 Bellaterra, Barcelona, Spain; belen.perez@uab.cat

**Keywords:** imidazolium salts, isocyanides, antiparasitic agents, *Trypanosoma brucei*, *Trypanosoma cruzi*

## Abstract

Imidazolium salts are privileged compounds in organic chemistry, and have valuable biological properties. Recent studies show that symmetric imidazolium salts with bulky moieties can display antiparasitic activity against *T. cruzi*. After developing a facile methodology for the synthesis of tetrasubstituted imidazolium salts from propargylamines and isocyanides, we screened a small library of these adducts against the causative agents of African and American trypanosomiases. These compounds display nanomolar activity against *T. brucei* and low (or sub) micromolar activity against *T. cruzi*, with excellent selectivity indexes and favorable molecular properties, thereby emerging as promising hits for the treatment of Chagas disease and sleeping sickness.

## 1. Introduction

Chagas disease and sleeping sickness are caused by infection with the protozoan parasites *Trypanosoma cruzi* and *Trypanosoma brucei* sp., respectively, and have been classified as Neglected Tropical Diseases. Sleeping sickness threatens millions of people in 36 countries in sub-Saharan Africa. Chagas disease is estimated to affect around 8 million people in endemic areas of 21 Latin American countries. The population at risk of these parasitic diseases normally has limited access to medical assistance, as they live in rural areas of developing countries. Moreover, due to immigration, war, and poverty, the control of infection can be complicated. Indeed, in recent years, an increasing number of Chagas disease cases have been reported in the US, European countries, and Canada [1,2].

Both infections are transmitted by insect vectors. In the case of Chagas disease, transmission can also occur by the congenital route, food contamination, blood transfer, and organ transplantation. Pathology in Chagas disease is mainly associated with the chronic stage, which occurs years after the initial infection, with cardiomyopathy being a common outcome. In African trypanosomiasis, second-stage disease—which occurs once parasites breach the blood brain barrier (BBB)—is the critical phase, being fatal unless treated. Due to serious side effects and other limitations of the currently available drugs, there is an urgent need to develop efficient medications against these diseases [3,4,5,6,7].

**Figure 1:**
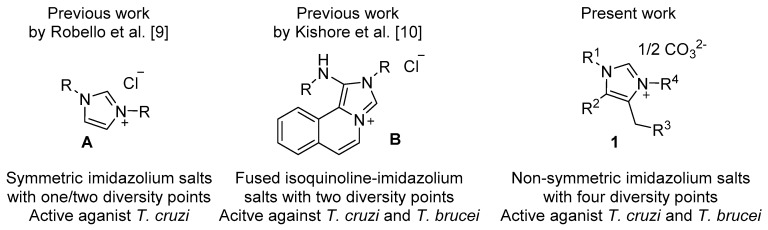
Structures of imidazolium salts active against *Trypanosoma* species: disubstituted imidazolium salts **A** [9], amino isoquinoline-imidazolium salts **B** [10], and the novel tetrasubstituted imidazolium salts **1**.

Imidazolium ions are valuable heterocyclic scaffolds due to their broad applications, especially in organocatalysis, and in the synthesis of liquid crystals. Recently, the biological activities of imidazolium and benzimidazolium ions has been reviewed, assessing their toxicity and particularly their wide range of uses in medicinal chemistry [8]. In 2014, Robello et al. [9] reported that simple symmetric imidazolium salts **A** (Figure 1) with bulky aromatic moieties on the N^1^ and N^3^ positions are active against *T. cruzi*. The putative biological target of these compounds has been postulated as being linked with the parasite mitochondrion. The major differences between these organelles in parasites and mammals could explain the selective toxicity of the imidazolium salts toward trypanosomes [8,9]. In this respect, we recently reported the potent antitrypanosomal activity of a family of azine-fused aminoimidazolium salts **B** (Figure 1) arising from a trimethylsilyl-catalyzed interaction of azines with isocyanides in a multicomponent manner [10]. Analogously, these compounds also presented two diversity points at the N atoms, and the most active hits displayed aliphatic residues at these positions.

Having developed a modular methodology for the facile synthesis of complex imidazolium salts [11], the goal of the present work was to study the antiparasitic activity of more complex unsymmetrical tetrasubstituted imidazolium salts **1** (Figure 1) against both African and American trypanosomiases. Relevantly, the target compounds feature a novel substitution pattern affecting not only the N atoms, but also at C atoms at positions 4 and 5.

## 2. Results and Discussion

### 2.1. Chemistry

**Scheme 1:**
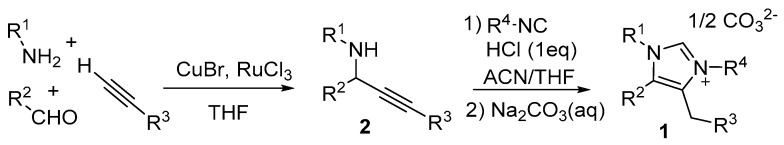
Synthetic access to the imidazolium salts **1**. ACN: Acetonitrile.

Compounds **1a**–**e** can be easily accessed through the novel two-step condensation of amines, aldehydes, terminal alkynes, and isocyanides [11]. First, the amine, aldehyde, and terminal alkyne substrates condense through a multicomponent A3 reaction to yield propargyl amines **2** in high yields [12]. In the second step, the formed propargylamine and isocyanide interact through an acid-catalyzed insertion-cyclization and isomerization process to give the desired imidazolium salts **1a**–**e** in good-to-moderate yields (Scheme 1) [11]. Interestingly, this two-step sequence can be performed in tandem. A similar process was reported by Zhu, featuring a dual catalytic system of AgOTf and Yb(OTf)_3_ [13]. The nature, scope, and commercial availability of the starting materials, the mild reaction conditions, and the modular access to imidazolium salts **1** (Table 1), with four diversity points, makes this synthetic approach ideal for medicinal chemistry, especially for hit finding through screening. In this respect, is relevant to comment that restrictions on the involved chemistry and the practical access to the alkyne inputs guided the initial screen on the substituent range at this initial stage. The A3 reaction with primary amines works better with anilines and aromatic aldehydes, and for these reason, R^1^ and R^2^ are substituted phenyl rings. On the other hand, there is a limited choice of commercially available alkynes; the most suitable/cheap are arylacetylenes (R^3^ being also phenyl or *p*-Me-phenyl), whereas there is a wider scope on R^4^ as many isocyanides (aliphatic, aromatic, functionalized) conveniently react in the second step of the sequence [11].

### 2.2. Biological Activity against T. brucei and T. cruzi

Next, we evaluated the trypanocidal activity of salts **1** against bloodstream forms of *T. brucei* and the epimastigote form of *T. cruzi* (Materials and Methods) (Table 2). Compounds **1a**–**d** displayed a potent bioactivity profile at low or sub-micromolar (*T. cruzi*) or nanomolar levels (*T. brucei*). For comparison, the EC_50_ value for nifurtimox—a drug used to treat *T. brucei* and *T. cruzi* infections—is approximately 2 µM for both parasites [14]. In contrast to compounds **1a**–**d**, salt **1e** was significantly less active, possibly due to its zwitterion character in physiological conditions, which could compromise its ability to cross biological membranes. Compounds **1b** and **1d** exhibited the highest potency against both parasites, being clearly more active against *T. brucei*. Interestingly, both compounds feature aromatic moieties on N^1^ and N^3^. The cytotoxicity of these compounds was evaluated using L6 rat myoblast cells (Materials and Methods). They displayed high selectivity indexes, particularly with *T. brucei*, suggesting good potential for use as antitrypanosomal drugs.

### 2.3. Molecular Properties of Compounds ***1***

A number of molecular properties are usually considered at early stages of the drug discovery process for the prediction of some important pharmacokinetic attributes. High oral bioavailability is a desirable feature for most drug candidates. According to the widely known Lipinski’s rules, good oral bioavailability can be expected for compounds with molecular weight below 500 daltons, calculated log P (as a measure of lipophilicity) below 5, and no more than five hydrogen bond donors and ten hydrogen bond acceptors [15]. Additionally, other molecular properties have been reported to influence oral bioavailability, and hence are also commonly taken into account in early-phase drug discovery. Thus, low molecular flexibility—measured by the number of rotatable bonds, with a cutoff value of 10, and low polar surface area, measured as the sum of surface contributions of polar atoms or as the sum of tabulated surface contributions of polar fragments (topological polar surface area or TPSA) [16], with a cutoff value of 140 Å^2^—are also delimiters of good oral bioavailability [17].

Brain penetration is also a desirable pharmacokinetic attribute for antitrypanosomal agents, purported to be effective in the late stage of sleeping sickness, when the *T. brucei* parasites invade the central nervous system (CNS). Brain permeation is usually determined experimentally through the well-known in vitro parallel artificial membrane permeability assay for blood-brain barrier (PAMPA-BBB) [18]. Of note, researchers from Pfizer recently disclosed an in silico method for the evaluation of the likeliness of a given compound to act in the CNS [19], which is based on the calculation of the so-called CNS multiparameter optimization (CNS MPO) algorithm [20,21]. This algorithm can be rapidly calculated from several of the above-mentioned molecular properties—namely, molecular weight, calculated log P, hydrogen bond donors, and TPSA, together with calculated log D and pK_a_ of the most basic center [20]. Compounds with CNS MPO desirability scores over 4 (in a scale from 0 to 6) tend to display favorable pharmacokinetic and safety attributes for CNS drugs.

To get insight into the potential oral bioavailability and brain permeability of the target compounds, we experimentally determined their PAMPA-BBB permeabilities through the method of Di et al. [18] and calculated the molecular properties that influence oral bioavailability and the CNS MPO scores, using the Molinspiration calculation software [22], the ChemAxon Marvin Suite 17.28 (for clog D (at pH 7.4) and pKa values), and a Pfizer’s Excel active table, which is available online [20] (Table 3). With regard to the experimentally determined PAMPA-BBB permeabilities (Pe), according to the limits established by Di et al. [18], compound **1e** is the only one with low BBB permeation (Pe < 1.89), probably as a consequence of its zwitterionic character at the pH of the assay, which would preclude its permeation by passive diffusion, whereas the Pe values of the rest of compounds are in the range of uncertain permeability (5.16 > Pe 10^−6^ cm s^−1^ > 1.89). However, Pe values of compounds **1a** and **1b** are interestingly close to the threshold for high BBB permeability (Pe > 5.16). Interestingly, CNS MPO scores close to the threshold for a full alignment of pharmacokinetic properties were found for most of our compounds (see Table 3 and Supplementary Information, SI), which further supports their usefulness in late-stage sleeping sickness. Moreover, we found that these compounds are compliant with Lipinski’s rules, and have TPSA values and a number of rotatable bonds (nrotb) that are favorable for a good oral bioavailability (Table 3).

## 3. Materials and Methods

### 3.1. Compound Preparation General Methods

*N*-(aryl)propargylamines **2** were prepared from the corresponding amines, aldehydes, and terminal alkynes by a modification of Li and Wei’s protocol [12]: In a Schlenk tube, 2.0 mmol (1.00 equiv.) of the aldehyde and 2.2 mmol (1.1 equiv.) of the amine were dissolved in 5 mL of THF under N_2_ atmosphere. The tube was then sealed and the mixture was heated at 60 °C until complete consumption of the aldehyde (≈2 h). Next, CuBr (30 mol %), RuCl_3_ (3 mol %), and the alkyne (2.4 mmol, 1.2 equiv.) were added. The mixture was stirred at room temperature (≈30 min), and then heated at 50 °C until complete consumption of the imine (≈24 h). Afterwards, the solution was cooled, poured into water (40 mL), and extracted with DCM (3 × 20 mL). The combined organic phases were washed with water (2 × 20 mL), dried (anh. MgSO_4_), and concentrated under vacuum. Purification by flash chromatography on silica gel (hexanes/AcOEt) afforded the corresponding propargylamines.

Tetrasubstituted imidazolium salts **1** were prepared from the corresponding propargylamines and isocyanides through the recent procedure reported by the group [11]: Propargylamine **2** (0.5 mmol, 1 equiv.) was dissolved in THF (1.5 mL) and ACN (1.5 mL) under N_2_ atmosphere. Next, the isocyanide (0.5 mmol, 1 equiv.) was added. A 4 M HCl solution in dioxane (125 μL, 1 equiv.) was added and the mixture stirred at room temperature until total consumption of the propargylamine (≈4 h). Next the reaction was quenched with saturated Na_2_CO_3_ aqueous solution (20 mL) and extracted with AcOEt (3 × 10 mL). The combined organic phases were dried (anh. MgSO_4_) and concentrated under vacuum to afford the corresponding imidazolium salts **1**. Analytically pure samples of the products were obtained by flash chromatography on silica gel (hexanes/EtOH 9:1 to 7:3 *v*/*v*).

For detailed protocols and compound characterization, see Reference [11] and the Supporting Information.

### 3.2. Biological Activity Determination

#### 3.2.1. *T. brucei* Culturing and Evaluation of Trypanocidal Activity

Bloodstream form *T. brucei* (strain 221) were grown as described previously [23]. Compound activity was determined by culturing parasites in the presence of different concentrations so that the levels which inhibited growth by 50% (EC_50_) and 90% (EC_90_) could be calculated. Logarithmically growing *T. brucei* were diluted to 2.5 × 10^4^ mL^−1^ and aliquoted into 96-well plates. Then, compounds were added at a range of concentrations and the plates incubated at 37 °C. Each compound was tested in triplicate. Resazurin was added after 48 h and the plates incubated for a further 16 h. The plates were then read in a Spectramax plate reader. Results were analyzed using GraphPad Prism.

#### 3.2.2. *T. cruzi* Culturing and Evaluation of Trypanocidal Activity

*T. cruzi* epimastigotes (CL Brener stain) were cultured at 28 °C [24]. Activity was determined using 96-well plate assays. Cultures were diluted to 2.5 × 10^5^ mL^−1^, aliquoted in triplicate at a range of compound concentrations, and incubated for 4 days. Resazurin was added and the plates incubated for a further 3 days, then read in a Spectramax plate reader as outlined above.

#### 3.2.3. Cytotoxic Activity against Rat Skeletal Myoblast L6 Cells

Cytotoxicity was assessed using microtiter plates following a previously described protocol [25]. Briefly, L6 cells were seeded at 1 × 10^4^ mL^−1^ in 200 μL of growth medium at different compound concentrations. The plates were incubated for 6 days at 37 °C and then 20 μL resazurin was added to each well. After a further 8 h incubation, fluorescence was determined using a Spectramax plate reader.

### 3.3. Cheminformatics Calculations

The Molinspiration package [22] was used to calculate the molecular properties (miLog P, topological polar surface area (TPSA), molecular weight (MW), number of hydrogen bond acceptors (nON), number of hydrogen bond donors (nOHNH, number of rotatable bonds (nrotb)), and number of violations of Lipinski’s rules (nviolations). The CNS MPO scores were calculated according to Reference [20]. For the full data, see the Supplementary Materials.

### 3.4. Determination of Brain Permeability: PAMPA-BBB Assay

The in vitro permeability (Pe) of the tested compounds and fourteen reference drugs through a lipid extract of porcine brain membrane was assessed by using a parallel artificial membrane permeation assay. Commercial drugs and the target compounds were tested using a mixture of phosphate-buffered saline (PBS): EtOH 70:30. The assay was validated by comparing the experimental permeability with the reported values of the reference drugs. A lineal correlation between experimental and reported permeability values of the fourteen commercial drugs was established (experimental Pe = 1.6374 × reported Pe − 1.3839; R^2^ = 0.9203). From this equation and the limits established by Di et al. [18] for BBB permeation, three ranges of permeability were considered: compounds of high BBB permeation (CNS+): Pe (10^−6^ cm s^−1^) > 5.16; compounds of low BBB permeation (CNS−): Pe (10^−6^ cm s^−1^) < 1.89; and compounds of uncertain BBB permeation (CNS±): 5.16 > Pe (10^−6^ cm s^−1^) > 1.89.

## 4. Conclusions

In conclusion, we have demonstrated that complex tetrasubstituted imidazolium salts are active antiparasitic agents against both African and American trypanosomiases. The remarkably high potency, good selectivity indexes, and favorable physicochemical/pharmacokinetic properties of these compounds make them promising hits for future studies. These properties can be further optimized by exploiting the several diversity points of the scaffold and its multicomponent origin. Moreover, the synthetic approach described here will facilitate the generation of new compounds and the assessment of structure-activity relationship SAR due to its modular nature.

## Figures and Tables

**Table 1:** Structural description of imidazolium salts **1**.

**Compd.**	**R^1^**	**R^2^**	**R^3^**	**R^4^**	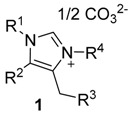
**1a**	*p*-Me-Ph	Ph	Ph	*tert*-Bu	
**1b**	*p*-MeO-Ph	*p*-Me-Ph	Ph	*p*-MeO-Ph	
**1c**	*p*-MeO-Ph	*p*-Cl-Ph	*p*-Me-Ph	*tert*-Bu	
**1d**	*p*-Me-Ph	*p*-Cl-Ph	Ph	2-naphthyl	
**1e**	*p*-Me-Ph	Ph	Ph	-COOH	

**Table 2:**
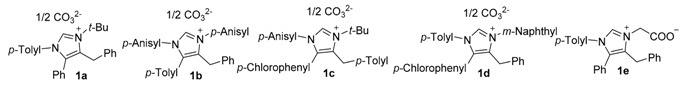
Biological activity of compounds **1** vs. bloodstream form *T. brucei* and *T. cruzi* epimastigotes.

	1a	1b	1c	1d	1e
**EC_50_** ^1^ (*T. brucei*, µM)	0.18 ± 0. 01	0.04 ± 0.00	0.18 ± 0.01	0.07 ± 0.00	5.26 ± 0.01
**EC_90_** ^2^ (*T. brucei*, µM)	0.28 ± 0.07	0.05 ± 0.00	0.20 ± 0.01	0.09 ± 0.00	6.79 ± 0.09
**EC_50_** (L6 cells, µM)	33.90 ± 3.30	4.60 ± 0.38	9.69 ± 0.58	4.09 ± 0.28	>130
**SI** ^3^ (*T. brucei*)	188	118	54	56	>24
**EC_50_** (*T. cruzi*, µM)	1.85 ± 0.29	0.44 ± 0.01	1.72 ± 0.39	0.54 ± 0.08	>20
**EC_90_** (*T. cruzi*, µM)	3.41 ± 0.14	1.29 ± 0.05	2.98 ± 0.19	1.00 ± 0.04	>20
**SI** (*T. cruzi*)	18	10	5.6	7.6	-

^1^ EC_50_: effective concentration to reduce parasite growth by 50%; ^2^ EC_90_: effective concentration to reduce parasite growth by 90%; ^3^ SI: Selectivity index; ratio of mammalian cell and parasite EC_50_ values.

**Table 3:** Molecular properties of compounds **1**.

	1a	1b	1c	1d	1e
**PAMPA-BBB (Pe)**	4.2	4.9	2.6	2.4	1.6
**CNS MPO**	3.3	3.5	3.7	1.4	5.8
**miLogP**	3.30	3.93	4.04	5.67	−1.35
**MW**	381.54	461.58	446.01	486.04	382.46
**nOHNH**	0	0	0	0	0
**nON**	2	4	3	2	4
**nviolations**	0	0	0	1	0
**TPSA**	8.82	27.29	18.05	8.82	48.95
**nrotb**	5	7	6	5	6

CNS MPO: CNS multiparameter optimization; MW: molecular weight; nOHNH: number of hydrogen bond donors; nON: number of hydrogen bond acceptors; nrotb: number of rotatable bonds; nviolations: number of violations of Lipinski’s rules; PAMPA-BBB (Pe): parallel artificial membrane permeability assay for blood-brain barrier permeability; miLogP: LogP calculated using Molinspiration; TPSA: topological polar surface area.

## Supplementary Materials

The following file is available online: a pdf file containing the experimental procedures for the synthesis of the compounds, their chemical characterization, additional data on the calculations, PAMPA and biological activity determinations.
