# Supplementary material for: Tetrasubstituted Imidazolium Salts as Potent Antiparasitic Agents against African and American Trypanosomiases
Source: Molecules. 2018 Jan 16;23(1):177. doi: 10.3390/molecules23010177 (PMC6017328; doi:10.3390/molecules23010177)
Supplement: Supplementary file 1 [file molecules-23-00177-s001.pdf]

Supporting Information

Tetrasubstituted Imidazolium Salts as Potent Antiparasitic Agents against African and American Trypanosomiases

Ouldouz Ghashghaei, Nicola Kielland, Marc Revés, Martin C. Taylor, John M. Kelly, Ornella Di Pietro, Diego Muñoz-Torrero, Belén Pérez and Rodolfo Lavilla

General information

Unless stated otherwise, all reactions were carried out under argon atmosphere in dried glassware. Commercially available reactants were used without further purification. Thin-layer chromatography was performed on pre-coated Merck silica gel 60 F254 plates and visualized under a UV lamp. ^{1}H and ^{13}C NMR spectra were recorded on a Varian Mercury 400 (at 400 MHz, and 100 MHz respectively). Unless otherwise stated, NMR spectra were recorded in $CDCl_3$ solution with TMS as an internal reference. Data for ^{1}H NMR spectra are reported as follows: chemical shift (δ ppm), multiplicity, integration and coupling constants (Hz). Data for ^{13}C NMR spectra are reported in terms of chemical shift (δ ppm). IR spectra were recorded using a Thermo Nicolet Nexus spectrometer and are reported in frequency of absorption (cm^{-1}). High resolution mass spectrometry was performed by the University of Barcelona Mass Spectrometry Service.

General procedure for the synthesis of propargylamines (2a-d)

N-(aryl)propargylamines **2** were prepared from the corresponding amines, aldehydes and terminal alkynes by a modification of Li and Wei's protocol [1]: In a Schlenk tube, 2.0 mmol (1.00 equiv.) of the aldehyde and 2.2 mmol (1.1 equiv.) of the amine were dissolved in 5 mL of THF under N_2 atmosphere. The tube then was sealed and the mixture was heated at 60 °C until complete consumption of the aldehyde (≈2 h). Next, CuBr (30 mol%), $RuCl_3$ (3 mol%) and the alkyne (2.4 mmol, 1.2 equiv.) were added. The mixture was stirred at room temperature (≈30 min), and then heated at 50 °C until complete consumption of the imine (≈24 h). Afterwards, the solution was cooled, poured into water (40 mL), and extracted with DCM (3 × 20 mL). The combined organic phases were washed with water (2 × 20 mL), dried (anh. $MgSO_4$) and concentrated under vacuum. Purification by flash chromatography on silica gel (hexanes/ AcOEt) afforded the corresponding propargylamines.

Characterization data propargylamines 2a-d.

Compounds **2a** and **2d** were previously described in the literature (See Refs. [2] and [3] respectively) and their properties matched with the reported data.

_N_-(1,3-diphenylprop-2-ynyl)-4-methylaniline (2a)

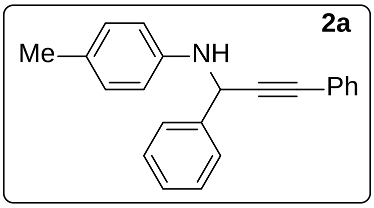

^{1}H NMR (400 MHz, $CDCl_3$) δ 7.58 (dd, J = 7.2, 0.8 Hz, 2H), 7.37 – 7.19 (m, 9H), 6.95 (dd, J = 8.6, 0.6 Hz, 2H), 6.65 (d, J = 8.4 Hz, 2H), 5.40 (s, 1H), 2.18 (s, 3H).). HPLC-MS (ESI): calculated for $C_{22}H_{20}N^+$ $[M+1]^+$: 298; found 298.

4-methoxy-_N_-(3-phenyl-1-_p_-tolylprop-2-ynyl)aniline (2b)

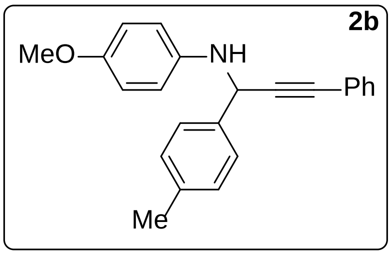

^{1}H NMR (400 MHz, $CDCl_3$) δ 7.55 (d, J = 8.0 Hz, 2H), 7.44 – 7.39 (m, 2H), 7.31 – 7.25 (m, 3H), 7.22 (d, J = 7.9 Hz, 2H), 6.84 – 6.74 (m, 4H), 5.38 (s, 1H), 3.76 (s, 3H), 2.38 (s, 3H). HPLC.-MS (ESI): calculated for $C_{23}H_{22}NO^+$ $[M+H]^+$: 328; found 328.

_N_-(1-(4-chlorophenyl)-3-_p_-tolylprop-2-ynyl)-4-methoxyaniline (2c):

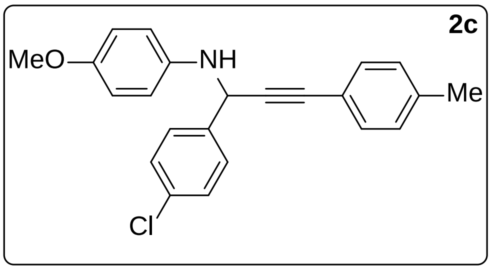

^{1}H NMR (400 MHz, $CDCl_3$) δ 7.59 (d, J = 8.3 Hz, 2H), 7.35 (d, J = 8.5 Hz, 2H), 7.29 (d, J = 8.1 Hz, 2H), 7.09 (d, J = 7.9 Hz, 2H), 6.82 – 6.71 (m, 4H), 5.38 (s, 1H), 3.75 (s, 3H), 2.34 (s, 3H). HPLC.-MS (ESI): calculated for $C_{23}H_{21}ClNO^+$ $[M+H]^+$: 362; found 362.

_N_-(1-(4-chlorophenyl)-3-phenylprop-2-ynyl)-4-methylaniline (2d)

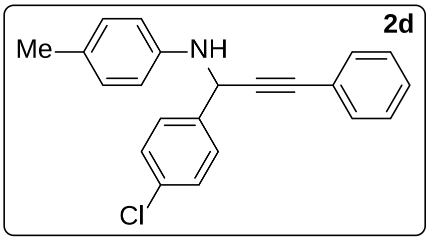

^{1}H NMR (400 MHz, cdcl$_3$) δ 7.59 (dd, J = 4.2, 3.6 Hz, 2H), 7.37 – 7.18 (m, 8H), 6.94 (dd, J = 11.4, 4.1 Hz, 2H), 6.66 (d, J = 7.1 Hz, 2H), 5.40 (s, 1H), 2.18 (s, J = 8.4 Hz, 3H). HPLC.-MS (ESI): calculated for $C_{22}H_{19}ClN^+$ $[M+H]^+$: 333; found 332.

General procedure for the synthesis of imidazolium salts (1a-e)

Tetrasubstituted imidazolium salts **1** were prepared from the corresponding propargylamines and isocyanides through the recent procedure reported by the group [4]: Propargylamine **2** (0.5 mmol, 1 equiv.) was dissolved in THF (1.5 mL) and ACN (1.5 mL) under N_2 atmosphere. Next, the isocyanide (0.5 mmol, 1 equiv.) was added. A 4 M HCl solution in dioxane (125 μL, 1 equiv.) was added and the mixture stirred at room temp. until total consumption of the

propargylamine (≈4 h). Next the reaction was quenched with saturated Na_2CO_3 aqueous solution (20 mL) and extracted with AcOEt (3 × 10 mL). The combined organic phases were dried (anh. $MgSO_4$) and concentrated under vacuum to afford the corresponding imidazolium salts **1**. Analytically pure samples of the products were obtained by flash chromatography on silica gel (hexanes/EtOH). Compounds **1a-e** were previously described in the literature and their properties matched with the reported data. See Ref. [4].

<u>Characterization data imidazolium salts 1a-e</u>

4-**Benzyl-3-(*tert*-butyl)-5-phenyl-1-(*p*-tolyl)-1*H*-imidazol-3-ium carbonate** (**1a)**

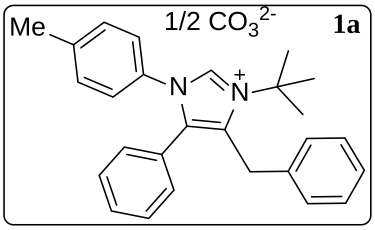

Following general procedure afforded imidazolium salt **1a** (from precursor **2a**) as clear brown oil. (Yield: 82%). ^{1}H NMR (400 MHz, $CDCl_3$) δ 9.67 (s, 1H), 7.28 – 7.24 (m, 3H), 7.23 (s, 2H), 7.19 (d, *J* = 1.1 Hz, 1H), 7.16 (dd, *J* = 8.4, 1.2 Hz, 2H), 7.08 (dd, *J* = 8.6, 0.5 Hz, 2H), 7.05 (dd, *J* = 8.3, 1.3 Hz, 2H), 7.01 – 6.95 (m, 2H), 4.24 (s, 2H), 2.24 (s, 3H), 1.68 (s, 9H). ^{13}C NMR (100 MHz, $CDCl_3$) δ 140.5, 136.8, 135.0, 131.3, 130.5, 130.4, 130.2, 129.8, 129.3, 129.1, 127.7, 127.4, 125.8, 125.4, 63.4, 31.2, 30.3, 21.3 (one quaternary carbon not detected). HRMS (ESI): calculated for $C_{27}H_{29}N_2^+$ $[M]^+$: 381.2325, found 381.2323.

4-Benzyl-1,3-bis(4-methoxyphenyl)-5-(*p*-tolyl)-1*H*-imidazol-3-ium carbonate (1b)

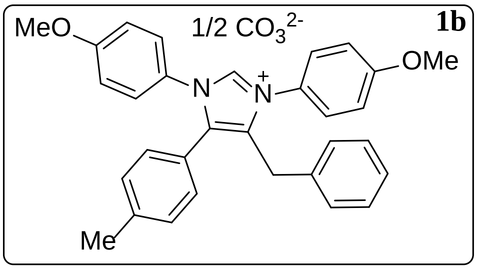

General procedure afforded imidazolium salt **1b** (from precursor **2b**) as brown solid. (Yield: 82%). ^{1}H NMR (400 MHz, $CDCl_3$) δ 8.89 (s, 1H), 7.45 (d, *J* = 9.0 Hz, 2H), 7.40 (d, *J* = 8.9 Hz, 2H), 7.22 (d, *J* = 8.2 Hz, 2H), 7.19 – 7.14 (m, 5H), 6.91 (dd, *J* = 8.9, 6.7 Hz, 4H), 6.81 – 6.76 (m, 2H), 4.00 (s, 2H), 3.84 (s, 3H), 3.81 (s, 3H), 2.34 (s, 3H). ^{13}C NMR (100 MHz, $CDCl_3$) δ 161.4, 160.9, 140.7, 135.9, 135.9, 133.2, 132.2, 130.6, 130.0, 128.9, 128.2, 128.2, 127.6, 127.3, 126.2, 125.5, 121.9, 115.1, 115.1, 55.8, 55.8, 29.5, 21.6. HRMS (ESI): calculated for $C_{31}H_{29}N_2O_2^+$ $[M]^+$: 461.2224, found 461.2227.

3-(*tert*-Butyl)-5-(4-chlorophenyl)-1-(4-methoxyphenyl)-4-(4-methylbenzyl)-1*H*-imidazol-3-ium carbonate (**1c**)

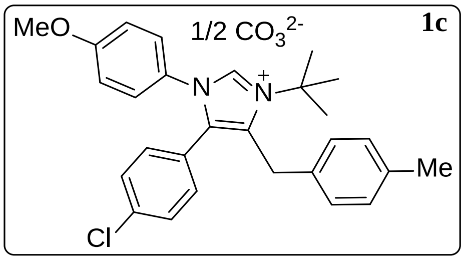

Using $MeSO_3H$ instead of HCl, afforded imidazolium salt **1c** (from precursor **2c**) as clear brown oil. (Yield: 56%). ^{1}H NMR (400 MHz, $CDCl_3$) δ 8.93 (s, 1H), 7.38

(d, *J* = 9.0 Hz, 2H), 7.20 (d, *J* = 8.6 Hz, 2H), 7.09 (m, 4H), 6.92 (d, *J* = 8.0 Hz, 2H), 6.87 (d, *J* = 9.0 Hz, 2H), 4.25 (s, 2H), 3.78 (s, 3H), 2.30 (s, 3H), 1.73 (s, 9H). ^{13}C NMR (100 MHz, $CDCl_3$) δ 160.8, 137.2, 136.6, 135.3, 134.4, 133.4, 132.0, 130.6, 129.9, 129.4, 127.8, 127.6, 126.2, 123.8, 115.0, 63.5, 55.7, 30.9, 30.2, 21.1, HRMS (ESI): calculated for $C_{28}H_{30}ClN_2O^+$ [M]$^+$: 445.2041, found 445.2045.

4-Benzyl-5-(4-chlorophenyl)-3-(naphth-2-yl)-1-(*p*-tolyl)-1*H*-imidazol-3-ium carbonate(1d):

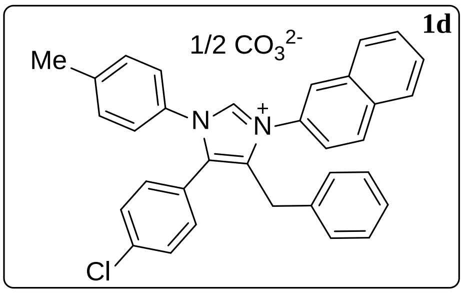

Following general procedure, afforded imidazolium salt **1d** (from precursor **2d)** as light brown oil. (Yield: 75%). ^{1}H NMR (400 MHz, $CDCl_3$) δ 9.65 (s, 1H), 8.21 (s, 1H), 7.94 – 7.83 (m, 3H), 7.70 – 7.52 (m, 5H), 7.34-7.26 (m, 4H), 7.23 (d, *J* = 7.5 Hz, 2H), 7.16 – 7.10 (m, 3H), 6.80 (m, 2H), 4.04 (s, 2H), 2.36 (s, 3H). ^{13}C NMR (100 MHz, $CDCl_3$) δ 141.0, 137.5, 136.9, 135.7, 133.8, 132.9, 132.3, 132.1, 131.7, 130.9, 130.7, 130.3, 130.2, 129.6, 129.0, 128.9, 128.3, 128.2, 128.0, 127.8, 127.4, 127.0, 126.2, 123.7, 123.3, 29.7, 21.4. HRMS (ESI): calculated for $C_{33}H_{26}ClN_2^+$ [M]$^+$: 485.1779, found 485.1782.

2-(4-benzyl-5-phenyl-1-(*p*-tolyl)-1*H*-imidazol-3-ium-3-yl)acetate (1e):

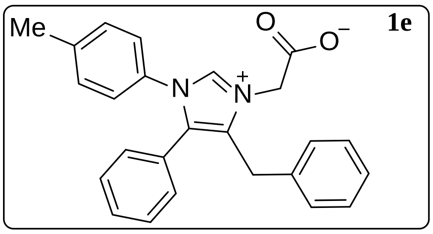

During flash chromatography to obtain the corresponding ethyl ester derivative (from precursor **2a**), imidazolium salt **1e** was also isolated as dark brown oil. (Yield: 11%) ^{1}H NMR (400 MHz, $CDCl_3$) δ 9.73 (s, 1H), 7.33 – 7.27 (m, 1H), 7.27 – 7.20 (m, 4H), 7.19 – 7.12 (m, 3H), 7.12 – 7.06 (m, 4H), 7.02 (d, *J* = 7.4 Hz, 2H), 4.69 (s, 2H), 4.05 (s, 2H), 2.25 (d, *J* = 1.9 Hz, 3H), one missing mobile proton. ^{13}C NMR (100 MHz, $CDCl_3$) δ 168.3, 140.4, 138.2, 135.5, 131.7, 131.4, 131.1, 130.5, 130.1, 129.3, 129.1, 128.0, 127.5, 125.6, 125.5, 50.9, 29.1, 21.3, one quaternary carbon not detected. IR (neat, cm^{-1}): 823.2, 1024.3, 1340.6, 1539.7, 1742.8, HRMS (ESI): calculated for $C_{25}H_{23}N_2O_2^+$ [M]$^+$: 383.1681, found 383.1679.

4-Benzyl-3-(*tert*-butyl)-5-phenyl-1-(*p*-tolyl)-1*H*-imidazol-3-ium carbonate (1a)

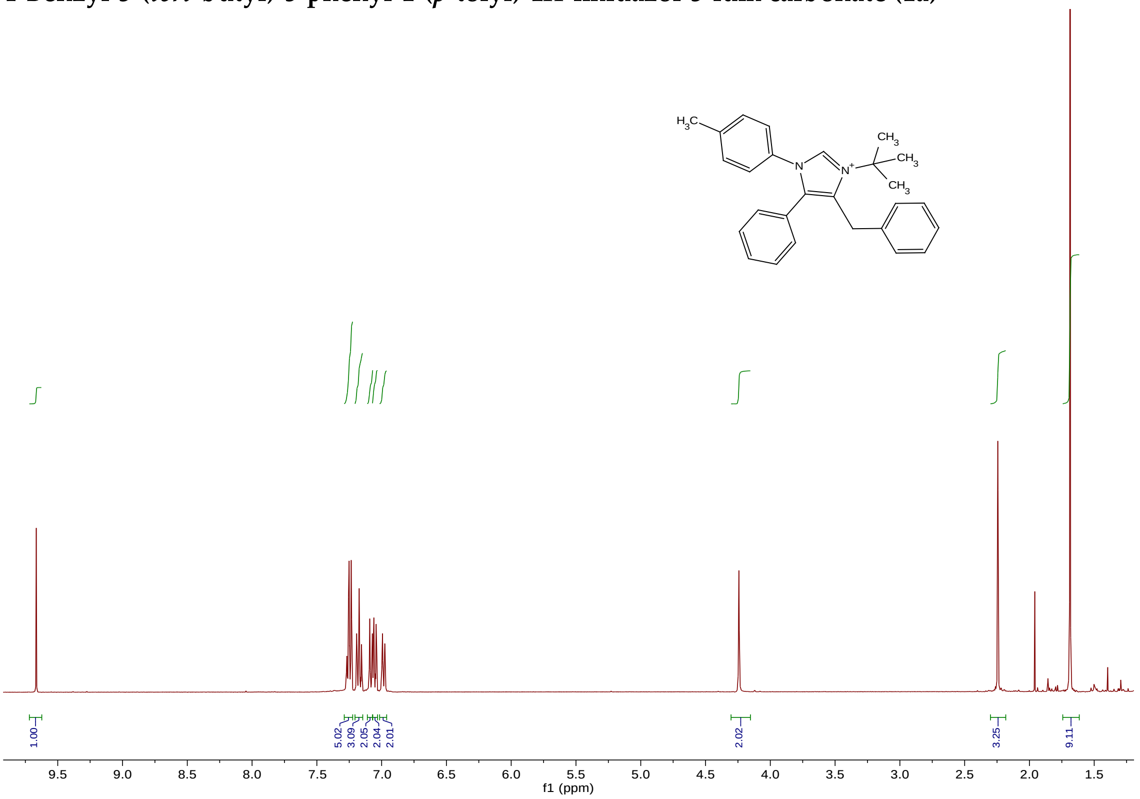

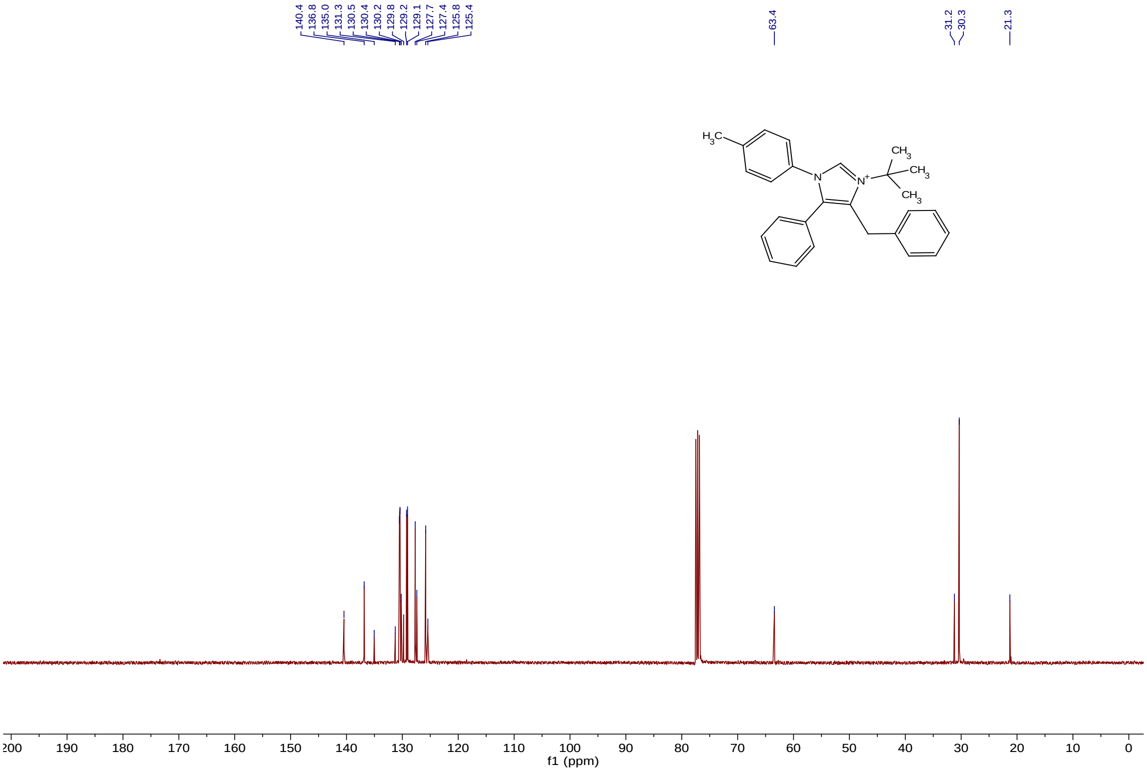

4-Benzyl-1,3-bis(4-methoxyphenyl)-5-(*p*-tolyl)-1*H*-imidazol-3-ium carbonate (1b)

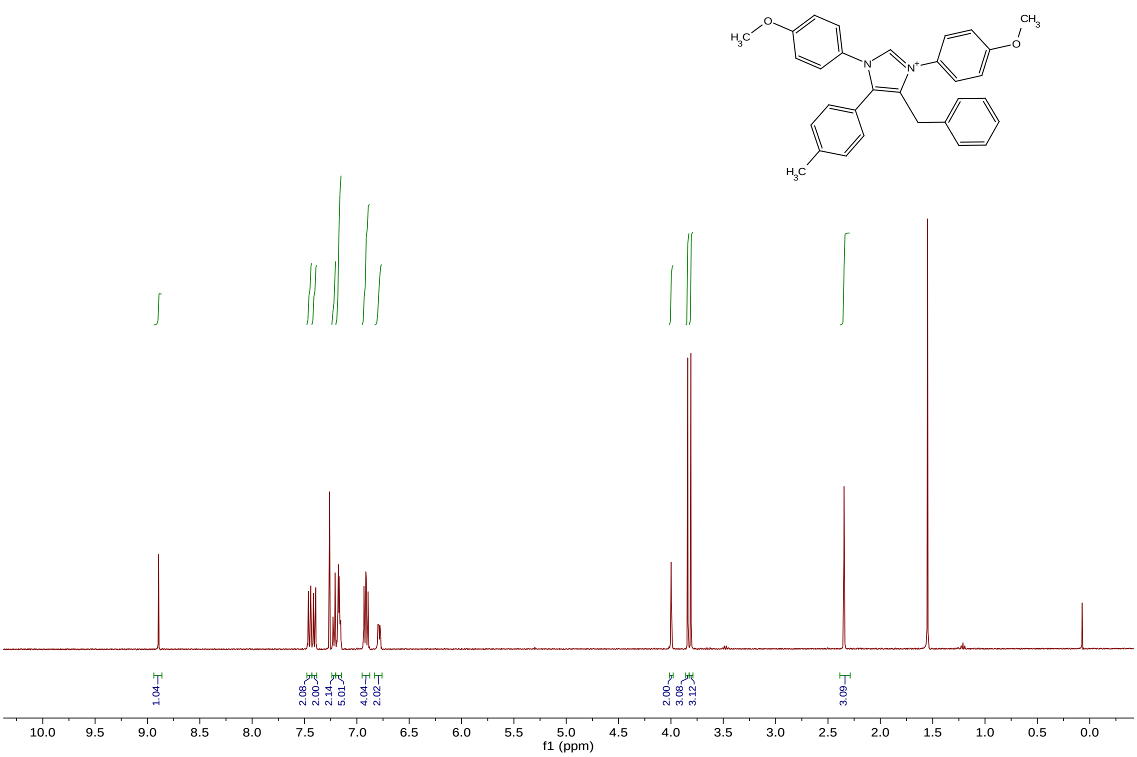

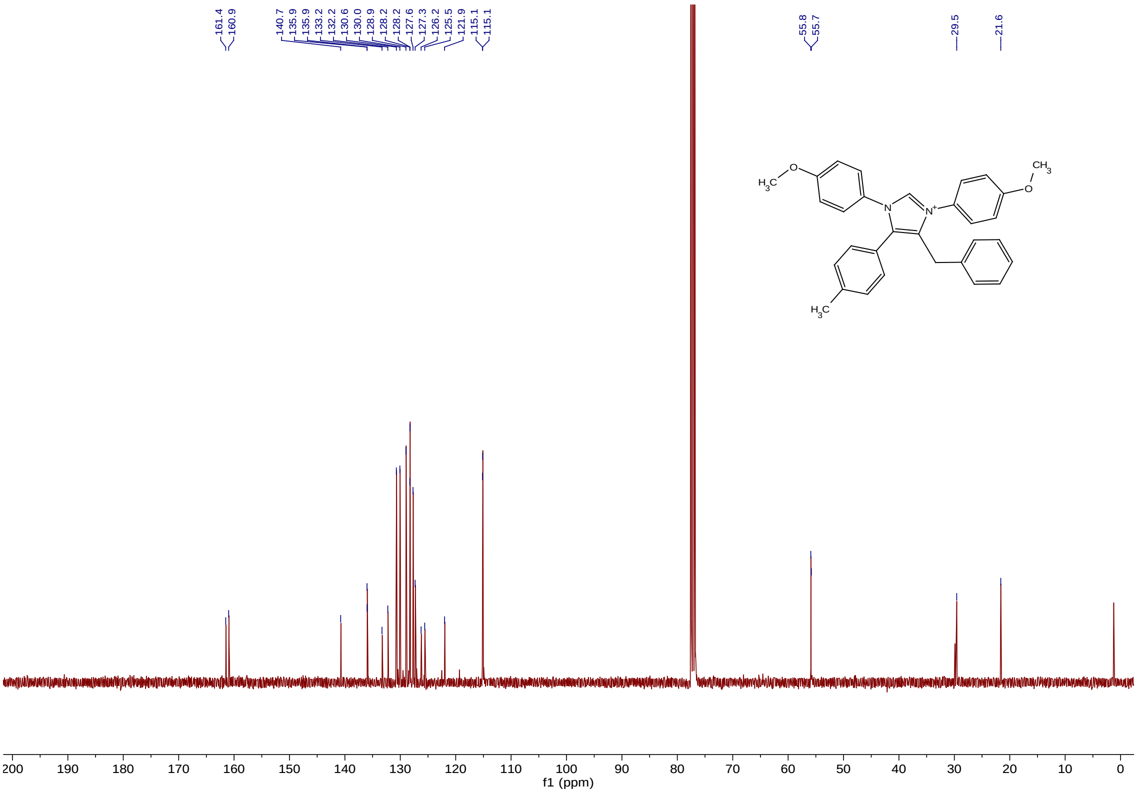

3-(*tert*-Butyl)-5-(4-chlorophenyl)-1-(4-methoxyphenyl)-4-(4-methylbenzyl)-1*H*-imidazol-3-ium carbonate (1c)

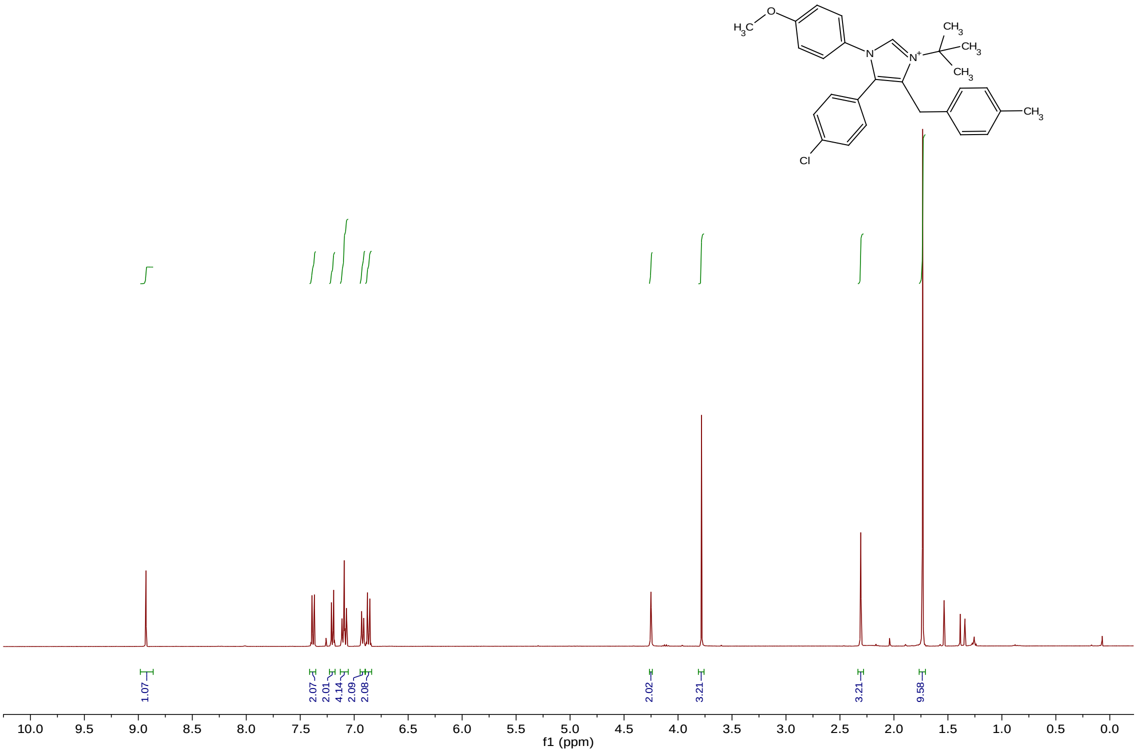

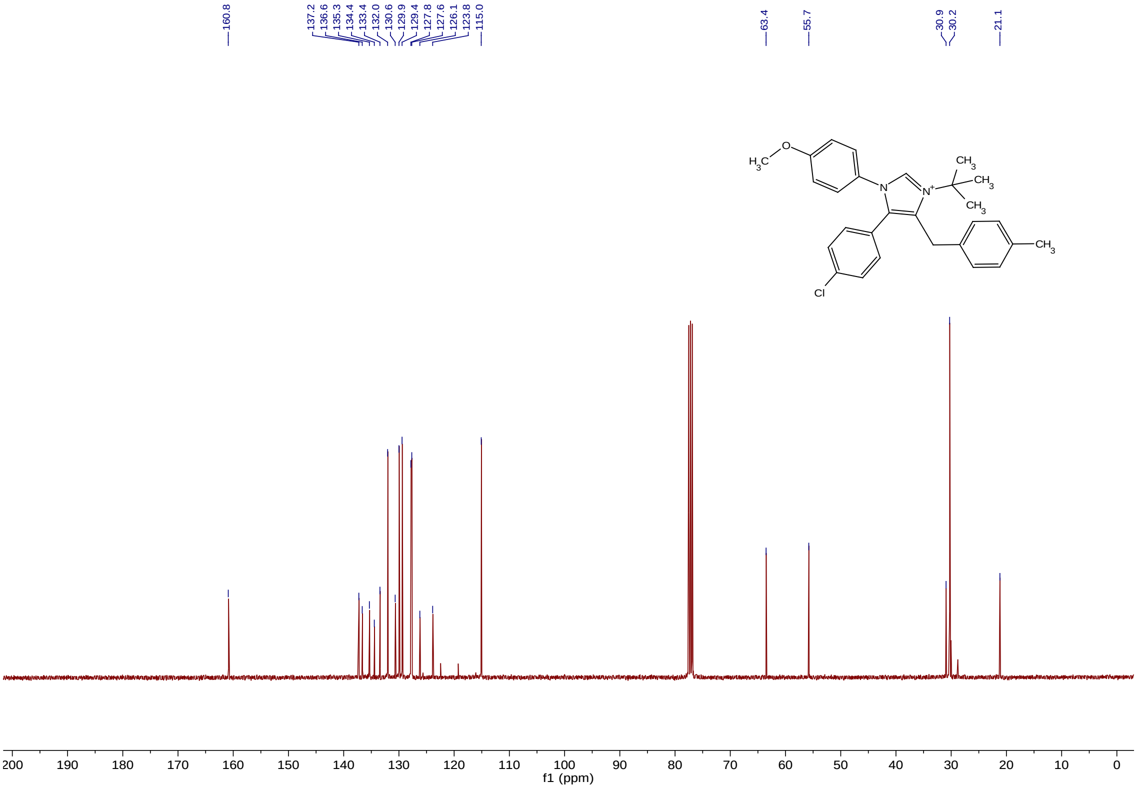

4-Benzyl-5-(4-chlorophenyl)-3-(naphthalen-2-yl)-1-(*p*-tolyl)-1*H*-imidazol-3-ium carbonate (1d)

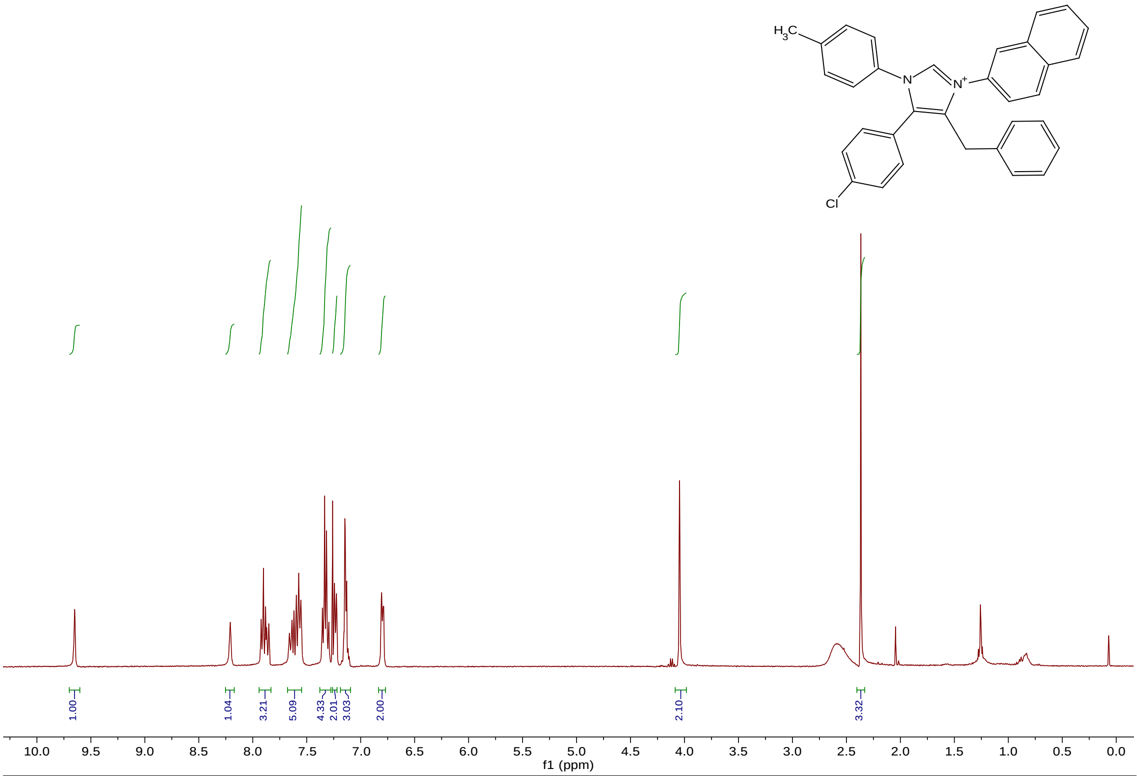

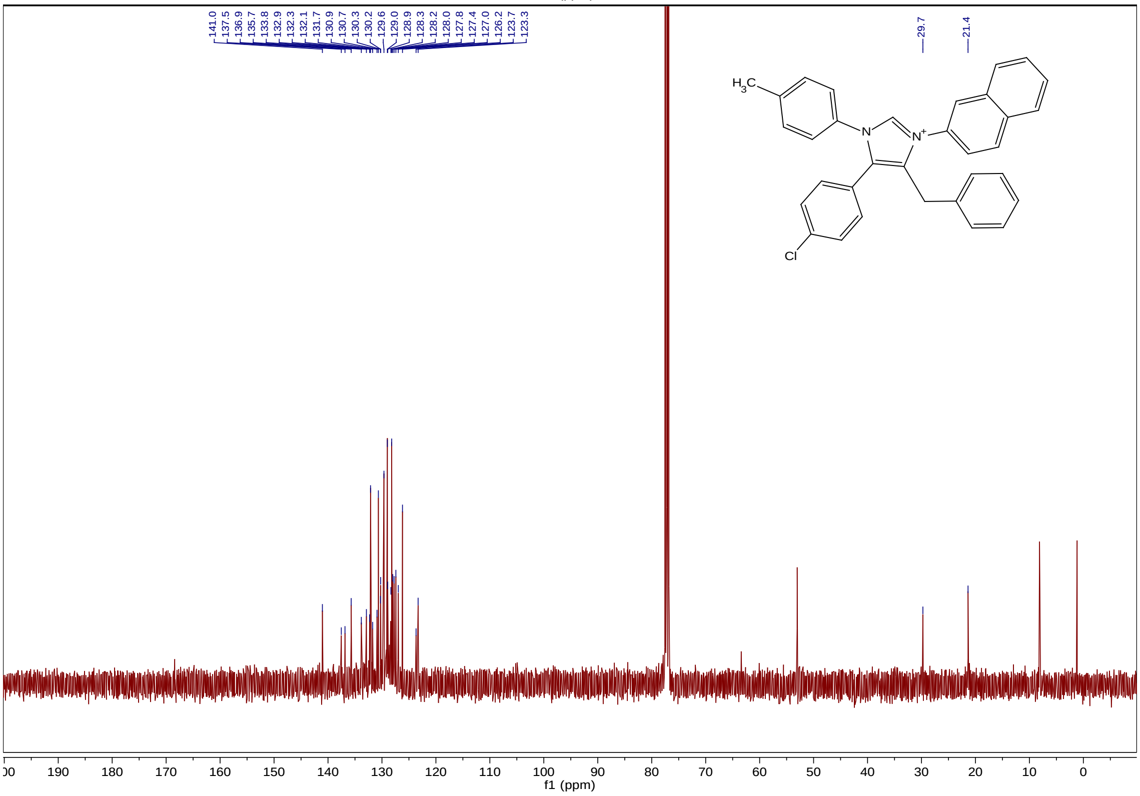

2-(4-benzyl-5-phenyl-1-(p-tolyl)-1H-imidazol-3-ium-3-yl)acetate (1e)

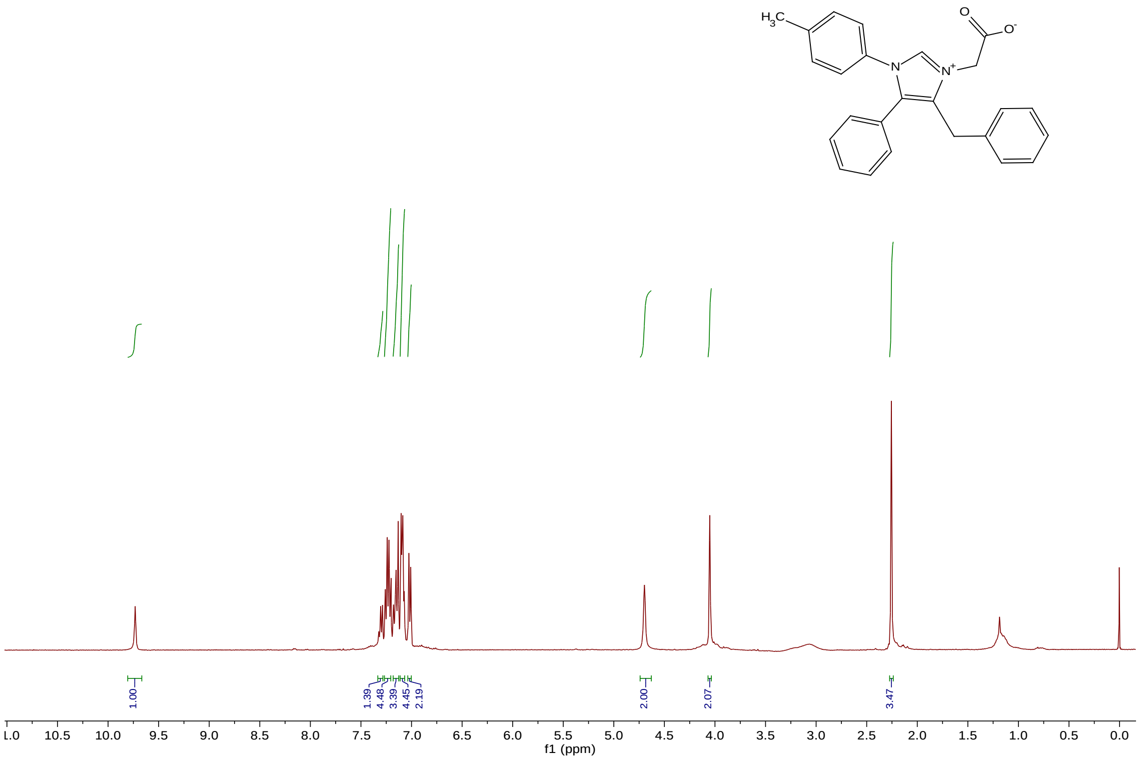

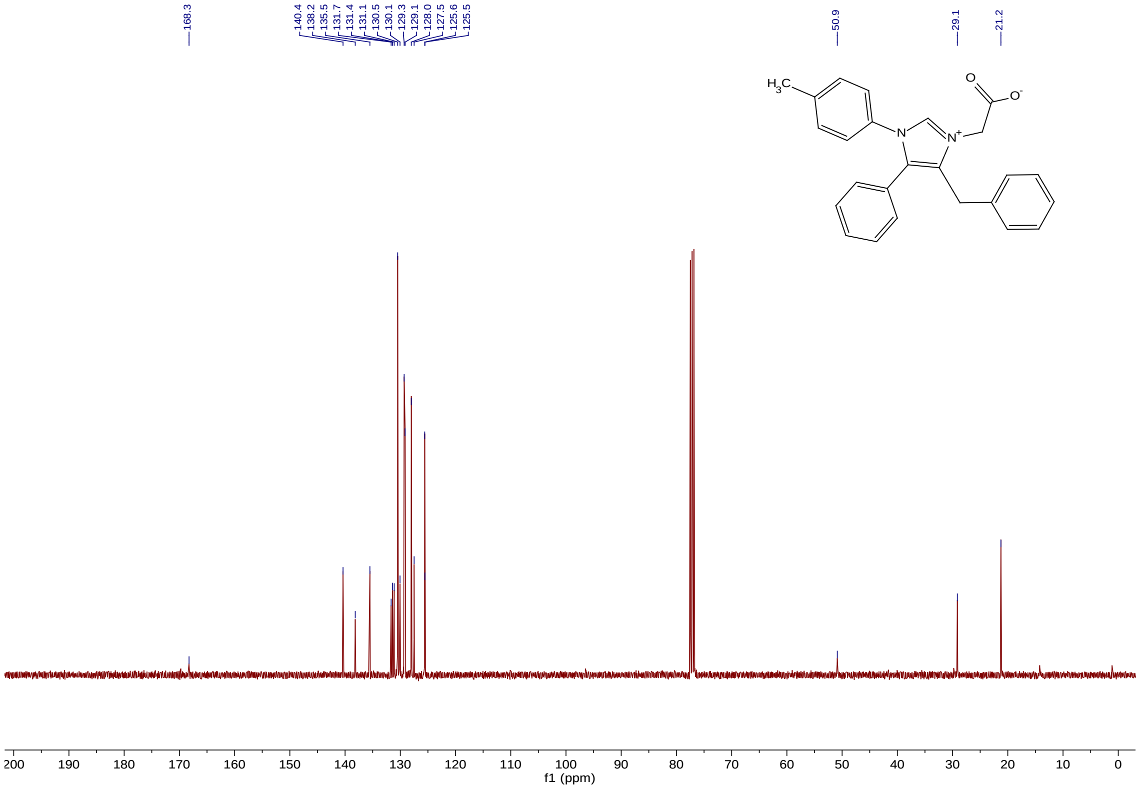

Biological Assays

Table S1. Compounds **1** vs bloodstream form *Trypanosoma brucei*.

Compd	EC_{50} *T. brucei* (μM)	EC_{90} *T. brucei* (μM)	EC_{50} L cells (μM)	S.I. [a]
**1a**	0.18 ± 0.01	0.28 ± 0.07	33.9 ± 3.30	188
**1b**	0.04 ± 0.00	0.05 ± 0.00	4.60 ± 0.38	118
**1c**	0.18 ± 0.01	0.20 ± 0.01	9.69 ± 0.58	54
**1d**	0.07 ± 0.00	0.09 ± 0.00	4.09 ± 0.28	56
**1e**	5.26 ± 0.01	6.79 ± 0.09	>130	>24

[a] S.I. Selectivity Index

Table S2. Compounds **1** vs *Trypanosoma cruzi* epimastigotes.

Compd	EC_{50} *T. cruzi* (μM)	EC_{90} *T. cruzi* (μM)	EC_{50} L cells (μM)	S.I. [a]
**1a**	1.85 ± 0.29	3.41 ± 0.14	33.9 ± 3.3	18
**1b**	0.44 ± 0.01	1.29 ± 0.05	4.60 ± 0.38	10
**1c**	1.72 ± 0.39	2.98 ± 0.19	9.69 ± 0.58	5.6
**1d**	0.54 ± 0.08	1.00 ± 0.04	4.09 ± 0.28	7.6
**1e**	>20	>20	>130	-

[a] S.I. Selectivity Index

Table S3. Molecular properties (Log P, topological polar surface area (TPSA), molecular weight (MW), number of hydrogen bond acceptors (nON), number of hydrogen bond donors (nOHNH), number of rotatable bonds (nrotb), molecular volume (of the cation), and number of violations of Lipinski's rules (n violations)) calculated using Molinspiration (http://molinspiration.com).

Compd	miLogP	TPSA	nON	nOHNH	nrotb	nviolations	vol	MW
**1a**	3.30	8.82	2	0	5	0	382.26	381.54
**1b**	3.93	27.29	4	0	7	0	438.57	461.58
**1c**	4.04	18.05	3	0	6	0	421.34	446.01
**1d**	5.67	8.82	2	0	5	1	445.01	486.04
**1e**	-1.35	48.95	4	0	6	0	357.13	382.46

Table S4. CNS MPO scores calculated using the algorithm reported [5]. TPSA values, MW, and the number of hydrogen bond donors (nOHNH), used in the algorithm, are shown also in Table S3

Compd	ClogP	clogD	TPSA	MW	HBD	pKa	CNS MPO
**1a**	3.30	2.82	8.82	381.54	0	14	3.3
**1b**	3.93	3.32	27.29	461.58	0	-4.53	3.5
**1c**	4.04	2.24	18.05	446.01	0	-4.8	3.7
**1d**	5.67	6.4	8.81	486.03	0	14	1.4
**1e**	-1.35	0.31	46.12	382.46	0	2.45	5.8

Table S5. Permeability (*Pe* 10^{-6} cm s^{-1}) in the PAMPA-BBB assay of 14 commercial drugs and tested compounds and predictive penetration in the CNS.

Compd	Bibliography value [1]	Experimental value(n=3) ± S.D.	CNS Prediction
**Verapamil**	16.0	25.9 ± 0.4	
**Testosterone**	17.0	23.9 ± 0.3	
**Costicosterone**	5.1	6.7 ± 0.1	
**Clonidine**	5.3	6.5 ± 0.05	
**Ofloxacin**	0.8	0.97 ± 0.06	
**Lomefloxacin**	1.1	0.8 ± 0.06	
**Progesterone**	9.3	16.8 ± 0.03	
**Promazine**	8.8	13.8 ± 0.3	
**Imipramine**	13.0	12.3 ± 0.1	
**Hydrocortisone**	1.9	1.4 ± 0.05	
**Piroxicam**	2.5	1.7 ± 0.03	
**Desipramine**	12.0	17.8 ± 0.1	
**Cimetidine**	0.0	0.7 ± 0.03	
**Norfloxacin**	0.1	0.9 ± 0.02	
**1a**		4.2 ± 0.3	CNS+/-
**1b**		4.9 ± 0.9	CNS+/-
**1c**		2.6 ± 0.1	CNS+/-
**1d**		2.4 ± 0.05	CNS+/-
**1e**		1.6 ± 0.03	CNS-

[1] Taken from Di et al. [6]

References

1. Li, C.-J. ; Wei, C. Highly efficient Grignard-type imine additions *via* C-H activation in water and under solvent-free conditions. *Chem. Commun.* **2002**, 268–269, DOI: 10.1039/B108851N.

2. Rubio-Pérez, L.; Iglesias, M.; Munárriz, J.; Polo, V.; Miguel, P. J. S.; Pérez-Torrente, J. J.; Oro, L. A. A bimetallic iridium (II) catalyst:[{Ir (IDipp)(H)} 2][BF 4] 2 (IDipp= 1, 3-bis (2, 6-diisopropylphenylimidazol-2-ylidene)). *Chem. Commun.* **2015**, *51*, 9860-9863, DOI: 10.1039/C5CC03296B.

3. Zhang, K.; Huang, Y.; Chen, R. A novel efficient method for synthesis of propargylamines via three-component coupling of aryl azide, aldehyde, and alkyne promoted by iron-iodine-copper(I) bromide *Tetrahedron Lett.* **2010**, *51*, 5463–5465, DOI: 10.1016/j.tetlet.2010.08.024.

4. Ghashghaei, O.; Revés, M.; Kielland, N.; Lavilla, R. Modular access to tetrasubstituted imidazolium salts through acid-catalyzed addition of isocyanides to propargylamines. *Eur. J. Org. Chem.* **2015**, 4383–4388. DOI: 10.1002/ejoc.201500502.

5. Wager, T.T.; Hou, X.; Verhoest, P.R.; Villalobos, A. Moving beyond rules: The development of a central nervous system multiparameter optimization (CNS MPO) approach to enable alignment of druglike properties. *ACS Chem. Neurosci.* **2010**, *1*, 435-449. DOI: 10.1021/cn100008c.

6. Di, L.; Kerns, E.H.; Fan, K.; McConnell, O.J.; Carter, G.T. High throughput artificial membrane permeability assay for blood-brain barrier. *Eur. J. Med. Chem.* **2003**, *38*, 223-232, DOI: 10.1016/S0223-5234(03)00012-6.
